# Use of Formaldehyde in Poultry Production for the Treatment of Litter, Hatching Eggs, Hatcheries, and Feed: An Updated Mini Review

**DOI:** 10.3390/toxics13111003

**Published:** 2025-11-20

**Authors:** Pedro Henrique Gomes de Sá Santos, Gabriel da Silva Oliveira, Concepta McManus, Vinícius Machado dos Santos

**Affiliations:** 1 Faculty of Agronomy and Veterinary Medicine, University of Brasília, Brasília 70910-900, Brazil; 2 Federal Institute of Brasília—Campus Planaltina, Brasília 73380-900, Brazil; 3 Center for Nuclear Energy in Agriculture (CENA), University of São Paulo, São Paulo 13416-000, Brazil

**Keywords:** bacterial control, *Escherichia coli*, formalin, hatchery eggs, natural alternatives, paraformaldehyde, poultry health, toxic products, *Salmonella* spp., sanitization

## Abstract

Bacteria such as *Salmonella* spp. are primarily transmitted through contaminated eggs and infected poultry; however, other routes, including the movement of personnel, vehicles, and lapses in biosecurity protocols, also play a significant role in their dissemination within poultry systems. Control of a wide range of microorganisms, including bacteria, is often carried out using chemical agents, such as formaldehyde, applied in its solid, liquid, or gaseous forms. Reports on the use of formaldehyde in poultry production date back more than a century. However, it continues to attract research interest due to growing concerns about bacterial resistance, embryotoxicity, occupational exposure, the generation of toxic byproducts, and the search for safer alternatives in poultry production systems. It remains widely used worldwide, but comprehensive and updated evaluations of its efficacy, toxicity, and risks to both poultry and workers are still limited. This review aims to synthesize the current knowledge on the use of formaldehyde in poultry production. Overall, the synthesis shows that formaldehyde remains an effective but high-risk sanitizer whose continued use in poultry systems requires rigorous control and monitoring protocols, and that the development and adoption of efficient and safer alternatives is recommended.

## 1. Introduction

**Figure 1:**
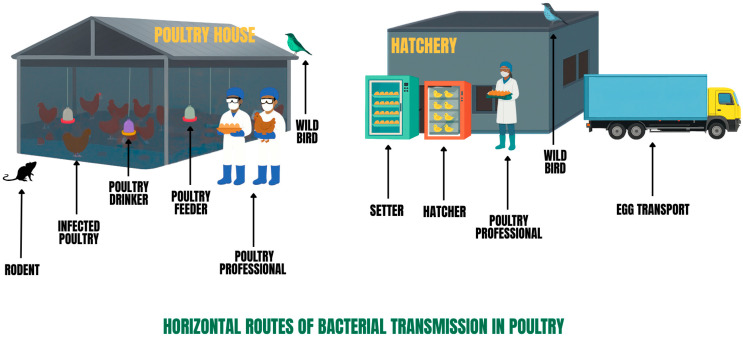
Horizontal routes of bacterial transmission in poultry systems. This figure illustrates some pathways by which pathogenic bacteria can spread within and between poultry facilities. In the poultry house, bacteria can be transmitted through direct contact with infected poultry, contaminated feeders and drinkers, and via fomites carried by poultry workers. Rodents and wild birds serve as mechanical vectors, introducing and disseminating bacteria within the poultry environment. In the hatchery, eggs and chicks can become contaminated during incubation in the setter and hatcher machines. Handling of eggs or chicks by poultry farmers, as well as the movement of vehicles transporting poultry products in and out of the facility, can further spread bacteria. Wild birds around the hatchery also pose a risk of introducing external bacteria.

*Salmonella* spp., *Campylobacter* spp., *Escherichia coli*, *Staphylococcus aureus*, and *Clostridium perfringens* are among the pathogenic bacteria commonly found in poultry systems, which can be spread through various routes of contamination, primarily via horizontal transmission [1,2] (Figure 1). These bacteria have impacted the poultry industry in the past, continue to do so today, and will likely become an even greater concern in the future. Colibacillosis and clostridiosis, caused by *Escherichia coli* and *Clostridium perfringens*, respectively, are major bacterial diseases that can lead to severe outbreaks, resulting in high mortality rates in poultry and substantial economic losses. This highlights the importance of prioritizing prevention and control in both broiler and laying hen production systems [3,4].

Bacterial control requires the effective application of sanitizers to achieve performance and quality goals in poultry production facilities. Formaldehyde is a sanitizing agent with strong antibacterial efficacy, ease of application, and low cost, making it readily available and straightforward to prepare and apply. However, strict safety measures are required due to its toxicity. These attributes have led to its global use in poultry production, and despite its long-standing use, it is still considered more advantageous than other sanitizers in the poultry industry. It is recognized as one of the most effective bactericidal agents for controlling bacteria in poultry operations in various countries [5,6,7,8]. It exhibits antibacterial activity against various bacteria, is cost-effective compared to other products, and is readily available in the poultry sanitizer market. Its use for bacterial control in poultry production has been associated with toxicity issues and may cause unintended impacts on the production environment [9]. This review aims to highlight the current knowledge of the effects of formaldehyde in poultry production environments, thereby contributing to an understanding of the interactions among formaldehyde, poultry, eggs, surfaces, and bacteria in hatcheries and farms.

For this purpose, publications investigating the use of formaldehyde in poultry production were identified through Google Scholar. Between 29 March 2025 and 7 November 2025, a bibliographic search was conducted in Google Scholar for research articles, reviews, books, and book chapters published in English, Portuguese, or Spanish, with no restrictions on publication date. The search strategy used different combinations of keywords related to formaldehyde, paraformaldehyde, formalin, toxicity, poultry, bacteria, contamination, infection, antibacterial, bacterial control, antibacterial resistance, laying hens, broilers, eggs, hatching eggs, embryo, chicks, sanitization, disinfection, sterilization, sanitizers, natural sanitizers, synthetic sanitizers, alternative sanitizers, disinfectants, synthetic disinfectants, natural disinfectants, chemical products, hatcheries, poultry workers and humans, employing the Boolean operators AND and OR. To complement the review, the sources of gray literature, such as conference papers, monographs, dissertations, and theses available online, were also consulted. For each search combination, the first ten results were screened based on their titles and abstracts to identify publications suitable for inclusion in the review. For those who passed this stage, the content of each selected study was critically analyzed. All works that met the criteria for the review, including language, thematic relevance, and alignment with the established topics and subtopics, were included. Studies that did not meet the objectives defined for each section were excluded, for example, in the topic “antibacterial effects”, this excluded those focusing exclusively on other microorganisms (such as fungi), as well as publications in languages other than English, Portuguese, or Spanish, duplicate articles, or those without full-text availability. Non-peer-reviewed materials were carefully evaluated by all authors, considering the credibility of the source, the clarity of the information, and its consistency with the evidence presented in peer-reviewed publications. Google Scholar was selected for its ability to integrate studies from different databases, a feature particularly relevant given the scarcity of research in this specific area. To maintain consistency in this review, the term “formaldehyde” has been used to encompass all its forms.

## 2. Formaldehyde in Poultry Field

Formaldehyde (CH_2_O) is the simplest aliphatic aldehyde, an electrophilic compound containing an aldehyde group (–CHO). At room temperature, it occurs as a colorless gas with a pungent odor. It can also exist as a white polymeric solid (paraformaldehyde) or as a colorless aqueous solution known as formalin (37–50% formaldehyde, often stabilized with 0–15% methanol) [10]. In aqueous media, formaldehyde is highly reactive and exists in dynamic equilibrium with methylene glycol (HO–CH_2_–OH) and its oligomeric forms (polyoxymethylene glycols). Its chemical reactivity arises from the electrophilic nature of the carbonyl carbon, which enables addition and condensation reactions with nucleophiles such as amines and phenols, as well as disproportionation under basic conditions (Cannizzaro reaction) to yield methanol and formic acid [10]. Formaldehyde can be applied by spraying (liquid dispersion), immersion (submersion in solution), or fumigation (vapor release in a closed space) [11]. In poultry production, formaldehyde can be applied in liquid form by spraying or in solid form. Most reports describe its use by fumigation, particularly for sanitizing hatching eggs, which remains the primary protocol in many countries, such as Brazil, Egypt, and Saudi Arabia. The success of fumigation depends on several factors, including environmental conditions, concentration, and exposure time. In this process, formaldehyde concentrations typically range from 5 to 14 g/m^3^, occurring at temperatures of 24 to 30 °C and relative humidity between 65 and 75%, resulting in exposure times of 15 to 40 min, depending on the study and experimental context [8,12,13,14,15,16,17]. These parameters act in combination, as temperature and humidity influence the gas’s activity, release, and diffusion, and together with concentration and exposure time determine its antibacterial effectiveness [9]. Additionally, effective containment of the gas within enclosed and well-sealed chambers is crucial to prevent leaks, maintain fumigation efficiency, and minimize occupational exposure risks [9,11]. The operational conditions can be adjusted according to the specific conditions of the farm or hatchery, ensuring maximum antibacterial efficiency while minimizing impacts on poultry, human, and environmental health.

Formaldehyde, regardless of its physical state, has a documented antibacterial potential [18,19,20]. It appears that the antibacterial potential of formaldehyde is related to alkylation reactions with cellular macromolecules [21]. Formaldehyde reacts with amino (–NH_2_), carboxyl (–C=O), hydroxyl (–OH), and sulfhydryl (–SH) groups of proteins, thereby disrupting disulfide bonds and altering their tertiary structure, as well as with purine bases in DNA and RNA [21,22]. Such covalent modifications ultimately result in bacterial inactivation. This mechanism of action, which has been reviewed and discussed for over 25 years, remains widely accepted and continues to be actively debated [22,23].

The main reason for the use of formaldehyde in industry is its antibacterial activity, characterized by its ability to reach inaccessible spaces, especially in protocols involving the release of the gas, similar to other antibacterial gases such as chlorine dioxide and ozone [15,24]. This allows for simultaneous bacterial control of materials, surfaces, and raw materials within a closed space. Antibacterial protocols involving formaldehyde have been used as one strategy in biosecurity programs aimed at interrupting reservoirs and horizontal transmission routes of pathogenic bacteria, such as those belonging to the Enterobacteriaceae family. These programs seek to reduce the dissemination of pathogens among poultry, between poultry and environmental surfaces, and between poultry products and humans, ultimately preventing disease outbreaks, such as salmonellosis, in the poultry industry. The potential spread of these bacteria beyond the production environment poses a risk to public health. For example, *Salmonella enterica* serovar Typhi can have a fatality rate of up to 80% in humans [25]. However, the likelihood of this scenario arising from poultry practices is minimal when sanitary standards, including those related to the use of formaldehyde, are correctly implemented and monitored.

### 2.1. Antibacterial Effects

**Figure 2:**
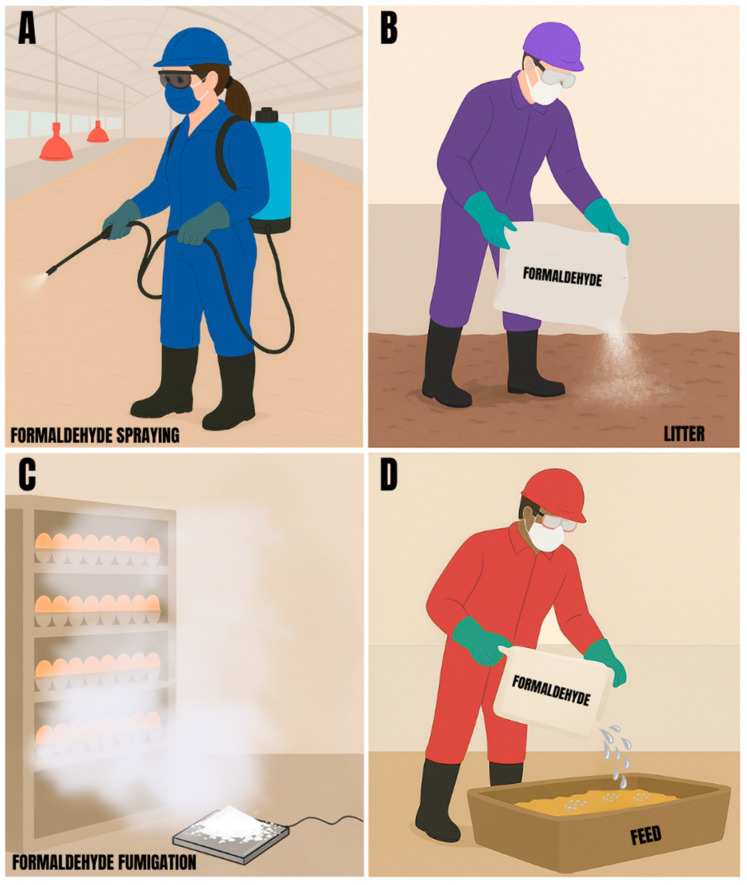
Some forms of formaldehyde application for bacterial control in poultry production, (**A**) house sanitization, (**B**) litter treatment, (**C**) hatching eggs fumigation and (**D**) incorporation of formaldehyde into feed.

In poultry practice, formaldehyde has been used beyond bacterial control in broiler and layer houses [26], with applications including the treatment of poultry litter, sanitization of hatching eggs and hatcheries, and even the incorporation into poultry feed (Figure 2).

#### 2.1.1. Poultry Litter

According to Toledo [27], the levels of *Pseudomonas* spp. and *Staphylococcus* spp. present in poultry litter were effectively controlled by the application of 15 g of 90% formaldehyde. After the first fumigation, *Pseudomonas* spp. counts dropped from 5.16 log_10_ CFU/g to undetectable levels within 24 h and remained undetectable throughout the 15-day evaluation period. Similarly, *Staphylococcus* spp. showed a marked reduction from 4.62 to 4.19 log_10_ CFU/g after 24 h, reaching only 2.03 log_10_ CFU/g after 15 days. Similarly, Argueta [28] reported that 8 g of formaldehyde (90–92%) can be used to eliminate coliform bacteria in poultry litter. Bampi et al. [29] experimentally evaluated the sanitization efficiency of 37% formaldehyde diluted to 4% in poultry litter contaminated with *Salmonella* spp. and *Escherichia coli*. After two hours of contact, formaldehyde significantly reduced *Salmonella* spp. by 1.4 log_10_ and *Escherichia coli* by 0.5 log_10_. Saleh et al. [30] mixed 5 g of 92% formaldehyde into the nest litter material and observed a significant reduction in the bacterial load. Total bacterial counts decreased from 8.28 to 7.70 log_10_ CFU/g after 24 h and to 7.59 log_10_ CFU/g after 72 h, increasing again to 8.11 log_10_ CFU/g after 120 h. Coliform counts declined from 8.18 to 7.55 log_10_ CFU/g after 24 h and to 7.23 log_10_ CFU/g after 72 h, with a slight increase to 7.30 log_10_ CFU/g after 120 h. According to the authors, this practice would likely decrease bacterial contamination of hatching eggs.

#### 2.1.2. Hatching Eggs

Luna [31] reported that fumigation with 2% formaldehyde achieved approximately 91% efficiency in reducing the total aerobic bacterial load on the eggshell surface. According to Rojas [32], fumigation with formaldehyde (11 mL/m^3^) removed approximately 90% of the organic matter on eggshells as measured by luminometry. Concurrently, microbiological analysis of the eggshells confirmed the elimination of *Escherichia coli*, *Klebsiella* spp., and *Pseudomonas* spp., demonstrating that formaldehyde can effectively remove organic matter that could otherwise protect microorganisms, thereby enhancing its sanitizing action on the eggshell surface against bacteria that naturally occur in poultry farms. Similarly, Vale et al. [33] demonstrated that applying a 1.5% formaldehyde solution (36.5–38% concentration) to eggs significantly reduced the total aerobic mesophilic bacterial count from 4.96 ± 0.52 to 2.03 ± 0.47 log_10_ CFU/mL, indicating a satisfactory reduction from a sanitary perspective. More recently, dos Santos et al. [34] estimated that fumigation with formaldehyde at a concentration of 6.07 g/m^3^ is effective in achieving minimal counts of mesophilic bacterial colony-forming units on the eggshell surface.

#### 2.1.3. Poultry Hatcheries

Harlia et al. [35] demonstrated that fumigation with a 40% formaldehyde solution eliminated bacterial contamination in the hatching airspace of poultry setters. This was also observed by Selby et al. [36], who conducted a controlled hatchery experiment in which 19-day-old embryonated eggs were exposed to a bacterial challenge designed to simulate natural contamination in commercial hatcheries. The inoculum, prepared from isolates of *Escherichia coli*, *Staphylococcus aureus*, and *Enterococcus* spp., was applied as a suspension containing approximately 10^7^–10^8^ CFU per 100 µL per egg over a 28 mm^2^ area located on the blunt end of the shell. Following this bacterial challenge, the treated hatchers were subjected to formaldehyde fumigation through drip application of 6 mL of formaldehyde every three hours, continuing until 12 h before hatching. The efficacy of formaldehyde was evaluated by quantifying Gram-negative bacteria, *Staphylococcus aureus*, and *Enterococcus* spp. recovered from the incubation environment (air and fluff samples) and from the gastrointestinal tract of newly hatched chicks. Air samples were collected using the open-agar plate method, in which selective agar plates were placed open on the top tray of the hatcher through a sampling port and exposed for 1 or 5 min, depending on the medium. In Experiment 1, for example, at approximately 80% hatch, airborne recovery of Gram-negative bacteria ranged from 1.62 to 2.16 log_10_ CFU/plate in the challenged or naturally contaminated groups, whereas formaldehyde treatment reduced this value to 1.11 log_10_ CFU/plate. Similar patterns were observed for *Enterococcus* spp. (1.73–1.86 versus 1.08 log_10_ CFU/plate) and *Staphylococcus aureus* (1.26–1.57 log_10_ CFU/plate versus undetectable), and the same trend occurred in Experiments 2 and 3. Formaldehyde fumigation significantly reduced the bacterial load across the other sampling sites evaluated, without affecting hatchability, seven-day body weight gain, feed conversion, and mortality. On the other hand, Espinosa [37] reported that fumigation of a commercial poultry hatcher, using a conventional system with 38% formaldehyde concentration (6.9 mL/m^3^), was insufficient to prevent an increase in the number of microorganisms in the air during the hatching period. Microbiological analysis of the down revealed that it was heavily contaminated.

#### 2.1.4. Feed Poultry

*Salmonella enterica* serovar Enteritidis can be eliminated from poultry feed within a short period of time after exposure to formaldehyde gas [38]. Avila et al. [39] reported that the feed for breeder hens containing 720 mg of formaldehyde per kilogram was free of *Salmonella* spp. Still, the initial levels of this pathogen were non-detectable in the untreated samples. The treated feed also had lower counts of total aerobic bacteria, Enterobacteriaceae, and *Clostridium perfringens*. The eggshells from these hens were also free of *Salmonella* spp. and showed reduced total aerobic bacterial counts.

The applicability of formaldehyde, due to its antibacterial activity, at various points along the production chain demonstrates that it can be adapted to diverse situations, making it a strategic differentiator for poultry production. This flexibility not only contributes to maintaining avian health but also supports the optimization of productive processes.

### 2.2. Bacterial Resistance to Formaldehyde

Aarestrup and Hasman [40] tested formaldehyde in vitro at concentrations ranging from 0.0008 to 0.4% against *Salmonella*, *Escherichia coli*, *Staphylococcus aureus*, *Staphylococcus hyicus*, *Enterococcus faecalis*, and *Enterococcus faecium*, which were isolated from broilers, cattle, and pigs. Most isolates exhibited minimal inhibitory concentrations ranging from 0.003 to 0.006%, with no significant differences observed among the bacterial species tested. Alijani et al. [41] tested formaldehyde at different concentrations, from a 37% stock solution, against Avian Pathogenic *Escherichia coli* isolates from broiler chickens. All strains exhibited minimal inhibitory concentration and minimal bactericidal concentrations at ≤0.009%. However, studies have shown that some bacterial strains can tolerate much higher levels of formaldehyde. Hoseinzadeh et al. [42] isolated *Escherichia coli* from cloacal and pericardial swabs collected from apparently healthy and colibacillosis-affected broiler chickens. The isolates were subjected to in vitro susceptibility testing using serial twofold dilutions of formaldehyde. According to the authors, isolates capable of growth at a concentration of 0.929% (9.29 mg/mL) formaldehyde were defined as resistant. Among the isolates, 15% were classified as resistant and 85% as sensitive to formaldehyde. Molecular screening revealed that 61% of isolates carried the class 1 integron, 94% harbored the formaldehyde dehydrogenase gene, and 8% contained the *orfF* gene.

Choroszy-Król et al. [20] prepared formaldehyde solutions from a 37% stock solution and tested them at concentrations of 0.02, 0.2, and 2.0% for 1 and 15 min against *Salmonella enterica* serovar Senftenberg and *Escherichia coli* isolated from poultry farms. The concentration of 2.0% completely eradicated the biofilms of all *Salmonella enterica* serovar Senftenberg strains after 1 min of exposure. At lower concentrations, 0.2 and 0.02%, only partial inhibition of bacterial growth was observed. The *Escherichia coli* strains showed higher tolerance, as some isolates were inactivated entirely by 2.0% after 1 min, while others with greater biofilm-forming ability required 15 min of exposure for complete eradication. Although formaldehyde concentrations of 0.2 and 0.02% produced some reduction in viable cell counts for certain *Salmonella enterica* serovar Senftenberg and *Escherichia coli* strains, these effects were strain-dependent and insufficient to ensure effective biofilm eradication. Brazilian researchers observed that all 63 avian pathogenic *Escherichia coli* isolates from poultry exhibited resistance to formaldehyde [43], underscoring the presence of formaldehyde-resistant bacteria in production environments.

Various studies have advanced our understanding of the bacterial molecular responses that confer resistance to formaldehyde. For example, Klein et al. [44] identified a novel formaldehyde resistance mechanism associated with the *yycR* gene, which encodes a zinc- and NAD^+^-dependent formaldehyde dehydrogenase. This enzyme, termed YycR, functions independently of thiols and can oxidize formaldehyde to formic acid, thereby contributing to cellular detoxification. The deletion of the *yycR* gene markedly reduced tolerance to formaldehyde, demonstrating that, in addition to previously recognized pathways mediated by 3-hexulose-6-phosphate synthase (*hps*), 6-phospho-3-hexuloisomerase (*phi*), and bacillithiol-dependent aldehyde dehydrogenase (*adhA*), an additional detoxification route based on YycR activity is also present.

Complementing these enzymatic detoxification mechanisms, Bazurto et al. [45] demonstrated that bacterial resistance to formaldehyde can also involve regulatory responses mediated by intracellular sensors. The study identified the protein EfgA (enhanced formaldehyde growth) as the first described bacterial formaldehyde sensor that does not act through enzymatic degradation of the compound. Instead, EfgA directly senses elevated intracellular formaldehyde levels and triggers immediate arrest of cell growth and protein translation, thereby preventing macromolecular damage caused by the aldehyde’s high reactivity with amino and thiol groups. In the same study, the authors also reported that, in addition to *efgA*, other genes identified through experimental evolution, such as *efgB* and hrcA, as well as genes involved in protein quality control, suggest that formaldehyde resistance is strongly associated with protection against protein damage rather than solely with chemical detoxification.

These molecular insights help explain the observed patterns of resistance across diverse bacterial species. For example, *Pseudomonas putida* strains can survive formaldehyde exposure through multiple protective mechanisms, including detoxification via glutathione-dependent formaldehyde and formate dehydrogenases that oxidize formaldehyde to CO_2_, efflux of the toxic molecule via the MexEF-OprN pump, and protection of cellular structures (DNA and proteins) [46]. Similarly, *Escherichia coli* may exhibit resistance to formaldehyde due to enzymes capable of degrading it into less toxic compounds, such as glutathione- and NAD^+^-dependent formaldehyde dehydrogenases encoded by a plasmid-mediated gene [47,48].

The prevalence of formaldehyde-resistant bacteria has significant consequences for poultry production. As reviewed by Oliveira et al. [49], *Escherichia coli*, for example, is a Gram-negative bacterium commonly isolated from eggs, dead-in-shell embryos, newly hatched chicks, and poultry. It is associated with septicemia, omphalitis, and congenital embryonic deformities, frequently resulting in high mortality rates. A survey conducted in just four Brazilian states revealed that contamination and diseases associated with pathogenic Escherichia coli result in million-dollar economic losses [50]. In this context, the bacterium’s increasing resistance to formaldehyde is particularly concerning, as *Escherichia coli* can be present at various points along the poultry production chain, where formaldehyde is widely used as a sanitizing agent. Therefore, a crucial point that cannot be overlooked is that the antibacterial efficacy of formaldehyde may be limited under certain application conditions. For example, Badr and Yoseif [26] reported that a 37% formaldehyde solution at a 10% concentration was unable to eliminate pathogenic bacteria in poultry houses completely. Moreover, the continuous use of formaldehyde in poultry production may favor the selection of resistant bacterial strains, such as *Pseudomonas aeruginosa* [51]. Therefore, careful planning of the protocol and continuous monitoring of process efficiency are essential.

### 2.3. Effects on Hatchability

The use of formaldehyde in poultry production is recognized for its low cost and ease of application, making it accessible to producers of various scales. Its application occurs primarily at points in the production chain preceding the hatching process, aiming to ensure high hatchability rates and optimize post-hatch productivity. For example, hatchability rates above 80% and no adverse effects after hatching on quality, mortality, weight gain, feed intake, or feed conversion ratio were observed in poultry originating from eggs sanitized by fumigation with a 40% formaldehyde solution [52]. Although there are reports evaluating post-hatch productive parameters, such as overall poultry performance, the primary production indicator associated with formaldehyde use remains hatchability. Reports comparing the hatchability of eggs fumigated with formaldehyde to that obtained with other sanitizing agents, such as ozone, hydrogen peroxide, and essential oils, are available (Table 1).

Badran et al. [54] demonstrated that treating hatching eggs with formaldehyde for 30 min, prepared from a mixture of 60 mL of formaldehyde, 30 mL of water, and 48 g of potassium permanganate, resulted in higher hatchability compared to 5% hydrogen peroxide. No significant differences in chick weight at hatch were observed between the two treatments. However, based on the analysis of blood components (total protein, albumin, globulin, glucose, triiodothyronine, uric acid, creatinine, aspartate aminotransferase, and alanine aminotransferase) and the relative organ weights, the authors suggested that chicks hatched from eggs fumigated with formaldehyde exhibited signs of hepatotoxicity, evidenced by hepatic hypertrophy and possible impairment of renal function.

Shahein and Sedeek [57] demonstrated that the hatchability of eggs fumigated with formaldehyde, obtained from a mixture of 119.8 mL formaldehyde and 59.9 g potassium permanganate per 2.83 m^3^ for 20 min, did not differ from that of eggs sprayed with a 14% propolis solution. Similarly, embryo weights at 18 days of incubation and at hatch were not significantly different between the two treatments. However, embryo and chick weights tended to be higher in eggs sanitized with propolis.

Oliveira et al. [56] reported that hatching eggs sanitized with essential oils of *Citrus aurantifolia* (9.38 mg/mL), *Ocimum basilicum* (4.69 mg/mL), and *Allium sativum* (1.17 mg/mL) showed superior hatchability performance compared to fumigation with formaldehyde, applied at a concentration of 5 g/m^3^ for 15 min. While embryos from eggs sanitized with essential oils showed no reports of tracheal or lung damage, embryos from eggs fumigated with formaldehyde exhibited tracheal lesions such as goblet cell hyperplasia, lymphocytic inflammation, and lung congestion. The weight of newly hatched chicks was comparable between the groups treated with essential oils and the formaldehyde group; however, formaldehyde significantly reduced chick weight compared with the non-sanitized control group.

Melo et al. [15] observed that the hatchability of fertile eggs sanitized with formaldehyde at a concentration of 5.03 g/m^3^ for 30 min was similar to that of eggs treated with ozone (5–15 ppm for 30 min), ultraviolet light at an average intensity of 8.09 mW/cm^2^ for 120 s, hydrogen peroxide at 3%, and peracetic acid at 0.3%. None of these sanitization methods harmed chick weight at hatching or the percentage of saleable chicks. From a production standpoint, formaldehyde can still be used to preserve hatchability potential, depending on the application conditions. This factor likely explains its continued use in poultry management despite growing pressure to replace it due to its high toxicity.

It is important to emphasize, however, that the apparent competitiveness of formaldehyde in maintaining hatchability rates should be interpreted with caution. Despite studies reporting hatchability performance similar to that observed with other sanitizers, such as hydrogen peroxide, ozone, or essential oils, these results are not entirely consistent (Table 1). In some cases, formaldehyde resulted in slight reductions in hatchability, while in others, it maintained or even exceeded the rates achieved with alternatives treatments (Table 1). This variability may be associated with differences in experimental protocols across studies, including concentration, exposure time, and even the quality and purity of the commercial product. Even though formaldehyde continues to show promise for sanitization and hatchability maintenance under specific controlled conditions, its efficacy must be evaluated contextually, accounting for methodological variability and, above all, the balance between antimicrobial efficacy and potential embryonic toxicity.

Formaldehyde is not exempt from causing reductions in hatchability under certain circumstances, as poultry are sensitive to toxic chemicals such as it. Its toxicity represents a central point of discussion within the scientific community [9,11,56,59]. The risks may extend beyond production losses, including the potential to compromise the quality of hatched chicks, the poultry’s healthy growth, and, above all, the safety of the professionals who handle and apply this chemical. Therefore, the hatchability results observed with formaldehyde are not universally guaranteed and may vary depending on management practices and experimental conditions. This emphasizes the need also to consider both its potential toxicity to embryos and the risks to poultry workers when making decisions about its use in poultry operations.

### 2.4. Toxicity in Poultry

Formaldehyde can cause a range of effects, from mild to severe outcomes, including poultry mortality, which occurs during both the embryonic phase and the post-hatch period. For instance, studies have shown that fumigation of hatching eggs with formaldehyde can lead to embryonic mortality rates exceeding 20% [60,61]. Additionally, other outcomes that may or may not progress to embryonic mortality have been reported depending on the study focus, encompassing fumigation of eggs and hatchery environments as well as poultry feeding, thus demonstrating varied impacts depending on the route, concentration and context of formaldehyde exposure [62,63,64].

These varied outcomes further demonstrate that poultry organs and tissues are particularly vulnerable to formaldehyde, exhibiting significant alterations across all developmental stages. The main observed effects were categorized by organ or tissue, with emphasis on the route of exposure (Table 2). In poultry, formaldehyde exposure can manifest as clinical signs such as apathy, depression, lethargy, anorexia, respiratory distress, and diarrhea, accompanied by reductions in body weight, weight gain, and feed intake, as well as changes in hematological and biochemical parameters [63,64,65]. Collectively, these manifestations reflect complex systemic impairments involving the liver, kidneys, heart, lungs, and gastrointestinal tract (Table 2), directly affecting the poultry’s clinical condition and growth performance.

**Figure 3:**
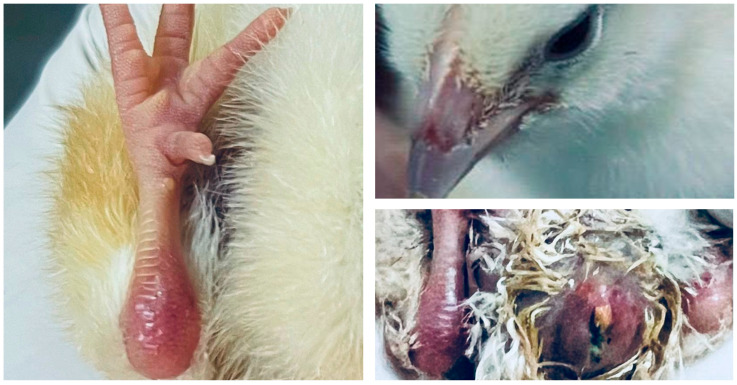
Morphological alterations observed in newly hatched chicks originating from eggs fumigated with formaldehyde in an experiment evaluating chicks from untreated eggs, eggs treated with formaldehyde, and essential oils. (**Left**) lesion on the hock, evidenced by swelling and erythema. (**Upper right**) lesion on the beak, characterized by areas of deformity. (**Lower right**) unhealed navel, presenting signs of omphalitis, with inflammation and exudate.

Several adverse outcomes in embryos and chicks originating from eggs fumigated with formaldehyde, as reported in studies published between 2015 and 2021, were summarized in a review by Oliveira et al. [11]. The concerning findings include embryonic anomalies, such as congenital malformations, reduced chick survival, and impaired chick quality. Chicks hatched from eggs sanitized with formaldehyde may exhibit compromised quality, evidenced by visible lesions, redness on the legs and beak, a swollen, edematous, and reddened abdomen, as well as poor navel healing (omphalitis) (Figure 3). In addition, severe eggshell wear, characterized by extensive structural damage and significant compromise, has also been reported [56]. The toxic effects of formaldehyde fumigation are related to its ability to penetrate the eggshell’s structure. The compound can react with structural components, such as proteins and minerals, and even reach the internal contents of the egg [9,71]. Consequently, toxic effects on embryos may occur even at relatively low concentrations, becoming progressively more severe with increasing concentration and exposure time [71]. In addition, its adverse effects may also be associated with the limited long-term antibacterial persistence, which favors subsequent bacterial recontamination of the eggshell surface and the embryonic development environment [71], allowing infectious processes to occur.

Valverde-Santiago and Pontel [72] reviewed the mechanisms involved in formaldehyde toxicity. They demonstrated that its harmful effects arise from its high reactivity with nucleophilic groups in DNA, RNA, and proteins, leading to the formation of adducts and crosslinks (DNA–protein, RNA–protein, and DNA–DNA). These lesions generate replicative and transcriptional stress, as well as single- and double-strand breaks, leading to genomic instability. Such damage activates multiple repair pathways, including the Fanconi anemia pathway, homologous recombination, nucleotide excision repair, and specific proteolytic mechanisms for the degradation of DNA–protein crosslinks mediated by the protease SPRTN and the proteasome. The review further highlights that formaldehyde reacts with glutathione, causing a redox imbalance and the accumulation of reactive oxygen species, and can inhibit enzymes involved in one-carbon metabolism, resulting in epigenetic dysregulation. At the cellular level, exposure to formaldehyde triggers ribotoxic stress and the integrated stress response through the formation of RNA–protein crosslinks, ultimately impairing translation and reducing cell viability.

The application of formaldehyde in poultry production, particularly through fumigation processes, can lead to the formation of toxic byproducts, including carbon monoxide and formic acid. These compounds have been associated with embryotoxic and oxidative effects in poultry. Matias et al. [73] observed that exposure of chicken embryos to carbon monoxide concentrations ranging from 3 to 18 ppm during the first 10 days of development resulted in a significant reduction in heart weight and a dose-dependent thickening of the ventricular, septal, and atrial walls, indicating that even very low levels of carbon monoxide may compromise embryonic cardiac development. Amini et al. [74] observed that exposure of chicken embryos to formic acid markedly reduced hatchability rates and significantly increased the levels of malondialdehyde, total lipid hydroperoxides, and total nitrite–nitrate, while decreasing total antioxidant capacity and superoxide dismutase activity in the liver, heart, and brain tissues. The authors concluded that formic acid, even at low concentrations (≥2 mM), can induce oxidative damage and embryolethality in avian embryos. Therefore, the potential conversion of formaldehyde into these reactive molecules during fumigation processes raises concerns regarding toxicology and biosafety. It has been reported that formic acid has limited use in poultry feed due to its corrosive nature toward the gastrointestinal tract [75]. Similarly, adverse effects associated with the inhalation of formic acid by animals have ranged from eyelid closure to mortality [76].

### 2.5. Risks for Poultry Workers

According to da Silva [77], the occupational exposure limit for formaldehyde allowed in Brazil is 2.3 mg/m^3^, while in other countries these values are considerably more restrictive, such as in Japan (0.24 mg/m^3^), Denmark (0.4 mg/m^3^), and Finland (1.2 mg/m^3^). Only the United States presents a slightly more permissive limit of 2.5 mg/m^3^. In contrast, the sanitization of hatching eggs frequently exceeds these limits by multiple times. In Brazil, for example, concentrations ranging from 5 to 13 g/m^3^ have been reported, approximately 2000 to 5000 times higher than the national occupational limit [8,13,14,56]. Although the procedure is conducted in closed fumigation chambers or rooms, the seal may not be absolute. Furthermore, exposure can occur intermittently and silently, especially during chamber opening, handling the product before fumigation, and manipulating newly fumigated eggs, creating toxic microenvironments. Under these conditions, local concentration may significantly exceed occupational safety limits. In the case of formaldehyde application to eggs using the spraying method, the procedure is usually performed by professionals.

The sanitization of hatching eggs with formaldehyde, typically performed by the worker during their daily 8 h shift, may continue over several working years. Additionally, these workers are often responsible for handling and applying this chemical to vehicles entering the farm and to the poultry houses [78]. This repetitive and prolonged exposure over many years can lead to chronic poisoning, causing severe neurological and respiratory damage, including cerebral atrophy, epileptic seizures, signs of dementia, as well as chronic rhinosinusitis with nasal septum deviation and perforation [79]. The intense routine and constant handling in environments where formaldehyde is manipulated and applied put the health and survival of workers at risk. Castellani et al. [80] reviewed the adverse effects of formaldehyde on human health, reporting bodily irritations, respiratory problems, allergic reactions, cancer risk, and even death.

### 2.6. Mitigating Formaldehyde Hazards in Poultry Systems

Mitigating formaldehyde hazards in poultry systems requires a combination of immediate protective measures, strict occupational protocols, and a progressive transition to safer alternatives. This involves the use of fully enclosed, automated fumigation systems, which control temperature and humidity, ensure adequate ventilation, isolate treated areas for a specified period, and properly dispose of product residues. It also requires adopting standardized protocols that define appropriate concentrations, exposure times, and application methods, based on research, manufacturer instructions, and regulatory guidelines. Proper worker training is essential to ensure safe handling [81], with a particular emphasis on the consistent use of personal protective equipment, including masks, gloves, goggles, aprons, and protective clothing. Occupational exposure limits vary widely between countries, from highly restrictive thresholds in Japan to more permissive values in Brazil and the United States [77]. Given that formaldehyde concentrations used in egg fumigation often exceed these limits by several orders of magnitude, aligning international regulations and enforcing stricter compliance are urgent priorities.

Promoting a transition to poultry farming that is less reliant on synthetic products, such as formaldehyde, and more focused on green alternatives, is the most effective way to prevent the harmful effects of this compound on poultry production and the professionals responsible for its management. As long as the intensive use of toxic synthetic products persists, ensuring the safety of poultry and humans will remain a significant challenge, both in large and small poultry systems. In the face of anticipated severely deleterious events, this challenge may, at some point in the future, lead to production failure. Positive gains should not be limited to productivity and economy alone, but should also encompass environmental safety, poultry health, and human health, ensuring that all these aspects are secured. For this reason, numerous studies have focused on developing and testing environmentally friendly, sustainable solutions for poultry systems. This list includes those tested and recommended for litter treatment, the sanitization of hatching eggs, and feed additives (Table 3).

Despite the availability of various green products, including those listed in Table 3, for routine poultry management practices, large-scale implementation of these compounds still faces obstacles that limit their official and widespread adoption in commercial production. Protocols involving essential oils and plant extracts often require adjustments to minimize limitations associated with high volatility, low solubility, instability, and variability in chemical composition. These characteristics directly affect production costs, which represent another limiting factor. The cost of using these natural compounds is usually higher than that of several synthetic sanitizers, such as formaldehyde, which reduces their economic feasibility, especially for small- and medium-sized producers. For this reason, recent research has focused on developing protocols using green products that employ minimal effective concentrations to reduce the amount required to achieve satisfactory antibacterial effects and, consequently, lower application costs. This approach is particularly relevant because some conventional chemical products require higher concentrations to achieve the same level of sanitization. Another significant challenge lies in defining standardized application protocols that ensure consistent antibacterial efficacy without compromising egg hatchability and/or poultry performance. In summary, large-scale applications still depend on improvements in formulation stability, cost reductions to levels comparable to those of synthetic products, and the establishment of standardized usage protocols.

## 3. Study Field Limitation

One of the major current challenges in the field of poultry production is the scarcity of detailed investigations into formaldehyde, despite its well-established role across different production sectors, both for its recognized antibacterial activity and its toxic potential. For instance, integrated research linking histopathology, genetics, and formaldehyde in poultry, as well as case studies involving professionals who handle this compound, is practically nonexistent. A few reviews have been published in recent decades, including one in 2009 that focused on egg sanitization with formaldehyde [9] and another in 2019 that addressed its incorporation into poultry diets [94]. However, it remains evident that the topic still needs to be explored in greater depth in the poultry field despite consistent efforts over the years. Among the numerous existing knowledge gaps are those related to bacterial resistance to formaldehyde in hatchery and farm environments, dose–response relationships associated with histological and genetic damage and malformations in poultry, residues and byproducts in poultry products, and the cumulative risks for workers exposed to fumigation vapors in poultry systems. Over the years, there has been a growing interest in alternatives to formaldehyde, as evidenced by the significant increase in publications on new sanitizers and substitute strategies. However, this focus on other options has essentially treated formaldehyde as a control treatment, without fostering innovative evaluations, resulting in reduced attention to its continuous and detailed study. Although limited in scope, the present mini review aims to update the current state of knowledge on the application of formaldehyde in poultry production, encouraging new investigations that more thoroughly explore the topics discussed herein.

## 4. Conclusions

The current literature indicates that formaldehyde has multiple applications in the poultry industry, ranging from its use as a feed additive to the sanitization of hatching eggs. This is due to its strong antibacterial efficacy, low cost, and practicality. When managed within well-studied and properly tested protocols, it remains an effective tool for controlling bacterial contamination in hatcheries and poultry farms. However, its well-documented toxicity to embryos, poultry, and workers raises significant safety concerns. The findings summarized here emphasize that the advantages of formaldehyde are closely linked to its proper handling and application, considering both concentration and exposure time. At the same time, its risks arise mainly from improper use and excessive exposure. Among the alternatives for antibacterial treatment in poultry production, oxidative sanitizers, such as hydrogen peroxide, and plant-based sanitizers, including essential oils and botanical extracts, are potential alternatives. Protocols involving these compounds need to be designed to minimize possible limitations and to suit each stage of the production chain, ensuring their routine use and meeting the demands of industrial-scale production.

## Figures and Tables

**Table 1:** Comparison of hatchability rates between formaldehyde and other sanitizers.

Formaldehyde	Ammonia	Ozone	Hydrogen Peroxide	Essential Oils	Propolis	Garlic Oil	*p* Value	Reference
Hatchability (%)								
45.00		40.00					ns	Souza et al. [17]
86.42		86.64	86.37				ns	Melo et al. [15]
81.87				84.69			ns	Oliveira et al. [8]
78.23		64.32	82.50				*p* < 0.05	Wlazlo et al. [53]
97.22			96.01				*p* < 0.05	Badran et al. [54]
91.10		91.79					ns	Hrnčár et al. [55]
75.34				87.83			*p* < 0.05	Oliveira et al. [56]
95.32					95.76		ns	Shahein and Sedeek [57]
93.46						94.07	ns	Rizk et al. [58]
93.1	93.5						ns	Cony et al. [13]

**Table 2:** Effects of formaldehyde exposure on organs of poultry, regardless of their developmental stage.

Structure	Observed Effects	Exposure Route	Reference
Cornea	Corneoscleral junction congestion Iris heterophil infiltration Basal cell degeneration	Hatchery fumigation	Espinosa [37]
Brain and head	Microcephaly Microphthalmia Exencephaly Brain degeneration Collapsed cavities Absence of segmentation Forebrain and hindbrain non-closure Severe ventricular reduction	Egg fumigation	Bekhet and Khalifa [66]
Spinal cord	Severe layer degeneration Central canal hemorrhage Central canal closure Zig-zag deformity Gray/white matter degeneration	Egg fumigation	Bekhet and Khalifa [66]
Trachea and lungs	Goblet cell hyperplasia Lymphocytic inflammation Deciliation Ciliary membrane rupture Ciliary agglutination Epithelial desquamation Heterophil infiltration Necrosis Congestion	Egg fumigation or hatchery fumigation	Di Matteo et al. [67], Oliveira et al. [56], de Freitas [68]
Proventriculus and Gizzard	Proventricular ulcers Hardened mucosa in both	Feed exposure	Khan et al. [64]
Liver	Congestion Sinusoidal dilation Minor hemorrhages Lymphoid infiltration Central vein congestion Tissue degeneration Reduced size	Poultry exposure or feed exposure	Khan et al. [64], Albaghdady et al. [69]
Kidneys	Atrophied glomeruli Ruptured glomeruli Leukocyte infiltration Tissue degeneration Hemorrhagic congestion Reduced size	Poultry exposure or feed exposure	Khan et al. [64], Albaghdady et al. [69]
Skeletal muscles	Breast hemorrhages Thigh hemorrhages	Feed exposure	Khan et al. [64]
Heart	Hemorrhage Congestion Inflammatory infiltration Reduced size	Feed exposure or poultry exposure	Khan et al. [64], Al-Saeed et al. [70]
Digestive tract	Lesions	Feed exposure	Khan et al. [64]
Reproductive system	Oviduct gland degeneration (nuclear vacuolation) Reduced ovary and oviduct size	Feed exposure	Khan et al. [64]

**Table 3:** Some green products to replace formaldehyde in poultry management.

Study Area	Green Product	Efficacy	Reference
Litter treatment	*Thymus vulgaris* essential oil	Reduced bacterial counts	Galgano et al. [82]
Litter treatment	Neem leaves	Improved poultry performance	Bishnoi et al. [83]
Litter treatment	Cinnamon essential oil	Bacterial reduction and the incidence of footpad lesions	Marchioro et al. [84]
Litter treatment	Plant parts of *Satureja hortensis*, *Origanum vulgare*, *Melissa officinalis*, *Salvia officinalis*, and *Thymus vulgaris*	Reduced bacterial counts	Gontar et al. [85]
Sanitization of hatching eggs	Tahiti lemon juice	Inhibited bacterial growth	de Jesus et al. [86]
Sanitization of hatching eggs	Cherry leaf extract	Improved hatchability rate	Ayuningtyas et al. [87]
Sanitization of hatching eggs	Tea tree and lavender essential oils	Reduced the bacterial load and improved hatchability	Iraqi et al. [88]
Sanitization of hatching eggs	Cemele pepper extract	Reduced the bacterial load and improved hatchability	Ergün et al. [89]
Feed additive	Coffee pulp extract	Improved growth performance and intestinal structure	Huanhong et al. [90]
Feed additive	*Curcuma xanthorrhiza* extract	Improved growth performance and intestinal microbial balance	Sinurat et al. [91]
Feed additive	*Minthostachys verticillata* essential oil	Improved growth performance and influenced the composition of the gut microflora, without inducing genotoxic or cytotoxic effects.	Escobar et al. [92]
Feed additive	Rosemary essential oil	Improved growth performance, nutrient digestibility, and carcass traits	Adil et al. [93]

## Data Availability

No new data were created or analyzed in this study. Data sharing does not apply to this paper.
